# Shewhart type simultaneous univariate control chart based on ranked set sampling scheme

**DOI:** 10.1016/j.mex.2025.103513

**Published:** 2025-07-16

**Authors:** Sukma Adi Perdana, Muhammad Mashuri, Muhammad Ahsan

**Affiliations:** a Department of Statistics, Institut Teknologi Sepuluh Nopember, Surabaya, Indonesia; b STAIN Sultan Abdurrahman Kepulauan Riau, Bintan, Indonesia

**Keywords:** Control charts, Max chart, Improved bootstrap control chart, Ranked set sampling

## Abstract

The control charts with the Ranked Set Sampling (RSS) scheme performs better in detecting shifts in mean and variance of the process compared to the control charts with the Simple Random Sampling scheme (SRS). In this article, we develop a Novel Shewhart type simultaneous univariate control chart (Max Chart) based on the RSS scheme. Since the distribution of the statistics $M(n_{i})$ based on the RSS scheme is not known, an alternative approach is taken by using an improved bootstrap method in developing a novel Shewhart type simultaneous control chart based on RSS. The results of the Monte Carlo simulation study show that, when compared to the conventional Max chart based on the SRS scheme, the average run length under out-of-control conditions (ARL_1_) obtained from that the Improved Bootstrap Max Chart based on the RSS scheme is superior in detecting shifts in mean and variance of the process. A real case example is also given to illustrate the operation and performance of the proposed control chart. Key points:

1. A novel Shewhart type simultaneous univariate control chart (Max Chart) based on the RSS scheme is proposed to improve the performance of the of control chart in detecting process shifts.
2. The Control limit of Shewhart type simultaneous univariate control chart (Max Chart) based on the RSS scheme was constructed using an Improved Bootstrap method.

## Graphical abstract

**Figure:**
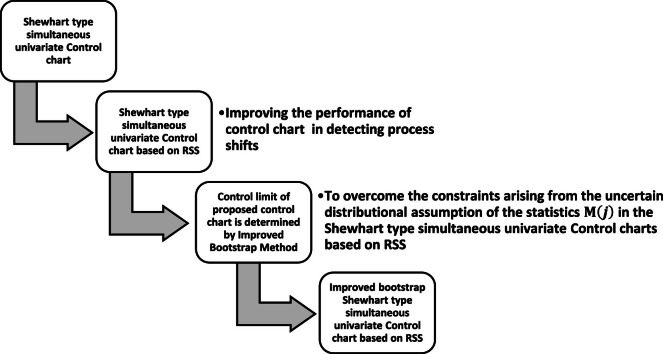

**Specifications table**

**Table:** 

**Subject area**	Mathematics and Statistics
**More specific subject area**	Statistics; Statistical Process Control
**Name of your method**	Shewhart type simultaneous univariate Control chart based on RSS
**Name and reference of original method**	G. Chen and S. W. Cheng, “Max *Chart: Combining X-Bar Chart And S Chart*,” Statistica Sinica, vol. 8, no. 1, pp. 263–271, 1998. S. A. Perdana, M. Mashuri, and M. Ahsan, “Improved bootstrap $\bar{X}$ control chart for non-normally distributed data,” MethodsX, vol. 14, p. 103,190, Jun. 2025, doi: 10.1016/j.mex.2025.103190.
**Resource availability**	Data will be made available on request.

## Background

One of the statistical methods commonly used in quality control is the control chart, which is useful for monitoring and detecting shifts in process parameters. The control chart was first developed by Shewhart in 1924 [1]. Based on the number of quality characteristics being monitored, control charts are divided into univariate and multivariate control charts. Univariate control charts were introduced by Shewhart [2], who developed the Shewhart-type control chart to monitor the Mean and Variability of processes [2]. Page in 1961 developed the Cumulative Sum (CUSUM) control chart to monitor the Mean of processes [3], Hawkins in 1981 developed the CUSUM type control chart to monitor the Variability of processes [4], Roberts in 1959 developed the Exponentially Weighted Moving Average (EWMA) control chart to monitor the Mean of processes [5], and MacGregor and Harris in 1993 developed the EWMA type control chart to monitor the Variability of processes [6].

Control charts can be divided into two categories based on the parameter being monitored: control charts for monitoring mean and control charts for monitoring variability. In monitoring the mean and variability, this can be done not only with separate control charts but also simultaneously using a single control chart. A control chart with characteristics like this is called a simultaneous control chart. Monitoring the process with a separate control chart is complicated and also impractical [7]. Therefore, simultaneous control charts are more practical and efficient compared to partial control charts. The simultaneous control chart not only can efficiently monitor the mean and variance simultaneously, but also the simultaneous control chart can still maintain its effectiveness to detect shifts in monitoring mean and variance. The Shewhart type simultaneous univariate control chart was first introduced by Chen and Cheng in 1998 and is known as the Max chart [8].

In statistical process monitoring, the performance of Shewhart type control charts can be improved by increasing the sensitivity of the control chart in detecting process shifts. The Shewhart type control charts using the Ranked Set Sampling (RSS) scheme perform better than The Shewhart type control charts based on the Simple Random Sampling (SRS) scheme [9]. The control chart with the Ranked Set Sampling (RSS) scheme was first introduced by Salazar and Sinha in 1997 [10], who developed a Shewhart-type control chart to detect shifts in the process mean. Subsequently, Muttlak and Al-Sabah in 2003 developed control charts to monitor the mean based on three schemes: RSS, median RSS, and extreme RSS [9]. Control charts based on these three schemes can detect shifts more effectively compared to control charts based on SRS. In 2019, Abbas et al. proposed a control chart for monitoring variability using the RSS scheme. The results showed that the control chart with the RSS scheme outperformed the control chart with the SRS scheme [11]. Hanandeh and Al-Nasser in 2020 developed a Shewhart control chart to monitor process mean by applying the Mini Max Ranked Set Sampling Scheme (MMRSS) [12]. Koyuncu and Karagoz in 2020 used the Neoteric Ranked Set Sampling (NRSS) scheme to develop a robust Shewhart-type control chart for monitoring the mean and range [13]. Noor-ul-Amin et al. in 2021 proposed a control chart to monitor the process mean under the Paired Double Ranked Set Sampling Scheme (PDRSS) [14]. Boroomandi and Kharrati-Kopai in 2022 proposed a non-parametric Shewhart control chart based on the Partially Rank-Ordered Set (PROS) scheme to monitor process means [15]. Next, Makhdoom and Basikhasteh in 2022 studied the efficiency of the control charts based on concomitant variable from the RSS scheme, Maximum Ranked Set Sampling with Unequal Samples (MRSSU), and Extreme Ranked Set Sampling (ERSS) for processes originating from the bivariate Farlie-Gumbel-Morgenstern distribution family [16]. Based on the previous explanation, The development of univariate Shewhart type control charts based on RSS has not yet addressed the simultaneous univariate Shewhart type control charts based on RSS. Therefore, in this article, we develop a simultaneous univariate control chart of the Shewhart type (Max Chart) based on the RSS Scheme.

To overcome the constraints arising from the uncertain distributional assumption of the statistics M(j) in the Max Chart based on the RSS, a non-parametric or distribution-free approach has been devised as a workaround. Several research have been undertaken to build control charts utilizing non-parametric or distribution-free approaches. A non-parametric method using the bootstrap procedure is one of the developments in this field. This method is preferred due to its proven track record in process monitoring, regardless of the assumed distribution [17]. In 1992, Bajgier introduced univariate control charts using the bootstrap method for estimating lower and upper control limits [18]. Seppala also developed a bootstrap control chart subgroup in 1995, which is generated using the residuals, or differences, between the mean of subgroup j and each observation value inside that subgroup j [19]. In 1996, Liu and Tang developed univariate control charts to monitor both independent and dependent observation data. These charts were developed utilizing bootstrap techniques [20]. For data with multivariate characteristics, the development of control charts using bootstrap techniques was first conducted to construct $T^{2}$control charts for monitoring observation data that is not normally distributed [17]. In 2020, a Bootstrap maximum multivariate CUSUM control chart was developed [21]. In this control chart, the bootstrap method is used to establish the control limits of the maximum multivariate CUSUM control chart [21]. The bootstrap method was also used by Kruba et al. in developing a multivariate simultaneous control chart of the Shewhart type as a tool to establish the control limits of the control chart [[21], [22], [23]]. Recently, Perdana et al. developed the Improved Bootstrap Control Chart, which performs more accurately than the Classic Bootstrap control chart [24]

In this paper, our focus is to propose the novel Max Chart based on the RSS using the improved bootstrap method. The control limit of the novel Max Chart based on the RSS is set based on the percentile of statistics $M(n_{i})$ obtained using the improved bootstrap method developed by Perdana et al., (2025). In order to evaluate the performance of the proposed control chart versus its competitor., the ARL_0_ and ARL_1_ of the Improved Bootstrap Max Chart based on RSS and the conventional Max Chart based on SRS were evaluated using Simulation.

## Method details

### Ranked set sampling

Ranked set sampling was first proposed by McIntyre in 1952 to estimate the average yield of agricultural land in Australia [25]. McIntyre utilized a concomitant variable from agricultural land to estimate the average yield of that land using the ranked set sampling (RSS) scheme. The basic concept of RSS is a collection of sampling units taken from a population that can be ordered using a concomitant variable without actual measurement of the desired variable, where measuring this variable is more costly and time-consuming [26,27].

The RSS method obtains sample units from a population that are more likely to encompass the entire range of values within the population. Thus, with the same sample size, the samples obtained through RSS are more representative than those obtained through SRS. The procedure for obtaining *h* sample units from the population using the RSS scheme is illustrated in Table 1. First, *h* sample units are randomly selected from the population and sorted according to their auxiliary variable. Various mechanisms can be used to determine the auxiliary variable of the observed units, which may include visual inspection or other quantitative variables but cannot involve measurements of the variable being observed.

**Table 1:** Illustration of the Sampling Procedure with the RSS Scheme.

Cycle 1	Cycle 2		Cycle c
X_[1]1_ ≤ *X*_[2]1_ ≤ *X*_[3]1_ ≤… ≤ *X*_[h]1_→**X**_[1]1_	X_[1]2_ ≤ *X*_[2]2_ ≤ *X*_[3]2_ ≤… ≤ *X*_[h]2_ →**X_[1]2_**		X_[1]_*_c_* ≤ *X*_[2]_*_c_* ≤ *X*_[3]_*_c_* ≤… ≤ *X*_[k]c_→**X_[1]c_**
X_[1]1_ ≤ *X*_[2]1_ ≤ *X*_[3]1_ ≤ … ≤ *X*_[h]1_→**X_[2]1_.**	X_[1]2_ ≤ *X*_[2]2_ ≤ *X*_[3]2_ ≤ … ≤ *X*_[h]2_ → **X_[2]2_.**	**…**	X_[1]_*_c_* ≤ *X*_[2]_*_c_* ≤ *X*_[3]_*_c_*≤ … ≤ *X*_[k]c_→**X_[2]c_**.
**.**	**.**		.
**.**	**.**		.
X_[1]_1 ≤ *X*_[2]_1 ≤ *X*_[3]1_ ≤ … ≤ *X*_[h]1_→**X_[h]1_**	X_[1]2_ ≤ *X*_[2]2_ ≤ *X*_[3]2_ ≤ … ≤ *X*_[h]2_→ **X_[h]2_**		X_[1]_*_c_* ≤ *X*_[2]_*_c_* ≤ *X*_[3]_*_c_*≤ … ≤ *X*_[h]c_ →**X_[h]c_**

The unit with the smallest order was selected as the first sampling unit. The first sampling unit is marked with X_[1]_, where square brackets [1] denote a ranking symbol. Then, the first-ranked unit is identified and taken for measurement X_[1]_, and the remaining units are discarded. Next, the sampling process as in the previous step is repeated, and the sample units are sorted again based on the accompanying variable; the second-ranked unit is taken for measurement X_[2]_, and the remaining units are discarded. This process was performed h times. The entire process produces h units of observation samples, which are measured as X_[1]_, X_[2]_,…, X_[h]_ and are referred to as one cycle. The total number of units *h* in each cycle is called the set size. The cycle was repeated *c* times to produce the desired n RSS samples with a sample size of *n=hc*.

The estimator of the mean parameter μ, denoted as $\hat{\mu }$ and the variability of the mean estimator is shown in equations as follows.

$$\hat{\mu }=\;\frac{1}{h}\sum_{i=1}^{h}\frac{1}{c}\sum_{j=1}^{c}X_{\left[i\right]j}=\frac{1}{h}\sum_{i=1}^{h}{\bar{X}}_{\left[i\right]}$$

$$Var\left(\hat{\mu }\right)=\frac{\sigma^{2}}{h}-\frac{1}{h^{2}}\sum_{i=1}^{h}{\left(\mu_{\left[i\right]}-\mu \right)}^{2}$$

$$Var\left(\hat{\mu }\right)=\;Var\left(\bar{X}\right)-\frac{1}{h^{2}}\sum_{i=1}^{h}{\left(\mu_{\left[i\right]}-\mu \right)}^{2}$$

The population variance estimator proposed by Stokes in 1980 [27] is as follows:

$${\tilde{\sigma }}^{2}=\frac{1}{hc-1}\sum_{i=1}^{h}\sum_{r=1}^{c}{\left(X_{\left[r\right]i}-\hat{\mu }\right)}^{2}$$

where

$$\hat{\mu }=\frac{1}{h}\sum_{i=1}^{h}\frac{1}{c}\sum_{j=1}^{c}X_{\left[i\right]j}$$

### The max chart control chart

The univariate simultaneous control chart (Max Chart) was developed by Chen and Cheng [8]. The main principle of the max chart is to transform the mean and standard deviation into a standard normal distribution *N*(0,1). The result of the mean transformation $\bar{X}$ is denoted U, and the result of the variance transformation S^2^ is denoted V. If the value of U or V exceeds the UCL, the process is out of control (OOC). The assumption that must be met to use the Max Chart is that the observed samples come from a normally distributed population.

Let

${\bar{X}}_{i}=\;\left(X_{i1}+\;X_{i2}+\;\ldots +\;X_{in_{i}}\right)/n$ be the mean of subgroup *i* and

$$S_{i}^{2}=\sum_{j=1}^{n_{i}}{\left(X_{ij}\;-\;{\bar{X}}_{i}\;\right)}^{2}/\left(n-1\right)$$

be the variability of subgroup *i*. Below are the variables U and V used in the Max Chart.

$$U_{i}=\;\frac{{\bar{X}}_{i}-\;\mu }{\sigma \;/\;\sqrt{n_{i}}}∼N\left(0,1\right)$$

$$V_{i}=\Phi^{-1}\left\{H\left(\frac{\left(n_{i}-\;1\right)S_{i}^{2}}{\sigma^{2}};n_{i}-1\right)\right\}$$

where

- Φ(z)=P(Z≤z) for Z∼N(0,1), standard normal distribution
- $\Phi^{-1}\left(\cdot \right)$ is inverse function of Φ(·)
- H(w;v)=P(W≤w|v) for $W∼\chi_{v}^{2}$ is chi square distribution with degree of freedom v.

Because $U_{i}$ and $V_{i}$ share the same distribution, central tendency, and dispersion, namely N(0,1), a control chart can be created using the statistic $M(n_{i})$ to monitor process mean and variability. The statistic $M(n_{i})$ is defined as follows.

$$M\left(n_{i}\right)=\;max\;\left\{|U_{i}\left|,\;\right|V_{i}|\right\}$$

The statistic $M(n_{i})$ above indicates that the value of $M(n_{i})$ is always non-negative, and the use of the maximum function is intended so that the simultaneous control chart that is formed can be used to monitor both the process center and process dispersion simultaneously.

The center line (CL) and upper control limits (UCL) of the Max Chart can be determined for various values of the first-type error probability, denoted as α. The values of CL and UCL depend on the value of α, as shown in Table 2.

**Table 2:** CL and UCL values in the Max Chart for the Type I error probability (α).

α	0.5	Α	0.0054	0.0027	0.00135
CL	1.0518	UCL	2.9996	3.2049	3.3994

### The proposed control charts

The Max Chart developed by Chen and Cheng was constructed using the SRS scheme [8]. In the simultaneous control chart or Max Chart based on the RSS scheme, the determination of the UCL values cannot fully follow Table 2 prepared by Chen and Cheng (1998) due to differences in the sampling scheme, which also results in different parameter estimators. To address this issue, this paper utilizes an improved bootstrap algorithm to determine the percentile of the statistic $M(n_{i})$ developed by Perdana et al., (2025). This step is performed to obtain an accurate UCL value for simultaneous Shewhart control charts using the RSS scheme.

### The improved bootstrap max chart control chart based on RSS scheme

This section discusses the procedures for constructing a Max Chart under the RSS scheme with the Improved Bootstrap method. The first step involves obtaining the sample with the RSS Technique. Step 2 involves determining the estimate values for the parameters $\mu_{rss}$, $\sigma_{{\bar{x}}_{rss}}$, and $\sigma_{rss}$. Step 3 involves transforming the mean and variance statistics into the conventional normal distribution. Step 4 is the procedure to determine the simultaneous statistics of the control chart. Step 5 is to determine the initial UCL value using the bootstrap technique. In Step 6, the initial UCL value is processed with the Improved algorithm to provide a final UCL value that corresponds to the data collected with the RSS technique. The detailed steps for constructing the Improved Bootstrap Max chart under the RSS scheme are as follows:

Step 1:

Take a sample of *n= hc* with the RSS scheme, where *h* is the number of samples in one cycle and *c* is the number of cycles or subgroups.

Step 2:

Determine the estimator for $\mu_{rss}$, $\sigma_{{\bar{x}}_{rss}}$, and $\sigma_{rss}$ from sample data taken with the RSS scheme.

$${\hat{\mu }}_{rss}={\overset{̿}{X}}_{rss}=\frac{1}{hc}\sum_{i=1}^{h}\sum_{j=1}^{c}X_{\left[i\right]j}$$

$$\sigma_{{\bar{X}}_{rss}}={\left[\frac{1}{h}{\hat{\sigma }}_{rss}^{2}-\frac{1}{h^{2}}\sum_{i=1}^{h}{\left({\bar{X}}_{\left[i\right]}-{\overset{̿}{X}}_{rss}\right)}^{2}\right]}^{1/2}$$

$${\hat{\sigma }}_{rss}^{2}=\frac{1}{hc-1}\sum_{i=1}^{h}\sum_{r=1}^{c}{\left(X_{\left[r\right]i}-\hat{\mu }\right)}^{2}$$

$$\hat{\mu }=\frac{1}{h}\sum_{i=1}^{h}\frac{1}{c}\sum_{j=1}^{c}X_{\left[i\right]j}$$

Step 3:

Determine the transformation equations $U_{jrss}$ and $V_{jrss}$ from the sample data estimator obtained using the RSS so that $U_{jrss}$ and $V_{jrss}$ follow a standard normal distribution.

$$U_{jrss}=\;\frac{{\bar{X}}_{jrss}-\;{\hat{\mu }}_{rss}}{\sigma_{{\bar{X}}_{rss}}}$$

$$V_{jrss}=\Phi^{-1}\left\{H\left(\frac{\left(n_{j}-\;1\right)S_{j}^{2}}{{\hat{\sigma }}_{rss}};n_{j}-1\right)\right\}$$

Step 4:

Determine statistics for control charts.

$$M\left(j\right)=\max\left\{\left|U_{jrss}\right|,\left|V_{jrss}\right|\right\}$$

Step 5:

Specify the initial UCL value using the bootstrap method according to the established first-type error probability, or α.the steps is explained as follows:

a. Generate $1000h^{2}$ data with a normal distribution $N({\hat{\mu }}_{rss},{\hat{\sigma }}_{rss})$.
b. Take a sample of 1000 subgroups with an RSS scheme, each containing h data.
c. Calculate 1000 M(j)statistics from the 1000 sample subgroups.
d. Select a size- n_b_ random sample with replacement from 1000 M(j) statistics.
e. Sort the sample statistics n_b_ bootstrap $M{\left(1\right)}^{*}$, $M{\left(2\right)}^{*}$,…, $M{\left(n_{b}\right)}^{*}.$
f. Determine the bootstrap upper control limit (UCL) which is ${\left(1-\alpha \right)}^{th}$ percentile of the ‘n_b_’ bootstrap sample statistics $M{\left(1\right)}^{*}$, $M{\left(2\right)}^{*}$,…, $M{\left(n_{b}\right)}^{*}$.
g. Proceed through steps d–f 10,000 times.
h. Calculate the initial UCL value, which is the mean of 10,000 Bootstrap UCL obtained from steps a to g.

Step 6:

The main purpose of the improved algorithm is to guarantee that the UCL obtained actually yields an ARL_0_ value of 370 for α=0.0027**.** This is performed by conducting the optimal point search for UCL, resulting in the optimal $\text{AR}L_{0}$ value. The bisection algorithm [[27], [28], [29], [30]] with δ and ε encourages the optimization process of the ARL value. The basic concept of this method is that the UCL values are shifted by δ to acquire UCL values that provide the optimal $\text{AR}L_{0}$ value with an error bounded by ε. The selection of δ and ε in the simulation depends on the magnitude of the control limit values to achieve the best optimization time. The steps of the improved algorithm are as follows:

a. Calculate the ARL_0_ value with the initial UCL value results from step 5.
b. Specify the value of δ and ε.
c. Specify UCL = UCL + δ and up = 0 if ARL_0__〈_ 370 Or UCL = UCL – δ, and up= 1 if ARL_0__〉_ 370.
d. Calculates the ARL_0_ value with new UCL.
e. Specify δ=δ/2 if $ARL_{0}$ < 370 and up = 1 and specify δ=δ/2 if $ARL_{0}$ > 370 and up = 0.
f. Stops the process if $|ARL_{0}-370|\leq \varepsilon$ and continues the process when $|ARL_{0}-370|>\varepsilon$.
g. Repeat steps c – f until $|ARL_{0}-370|\leq \varepsilon$ in order to obtain the best UCL.

## Method validation

### Simulation

In this section, evaluation methods are presented to compare the performance of the Max Chart with the SRS scheme and the Improved Bootstrap Max Chart based on the RSS scheme. The evaluation was based on the average run length value (ARL). In general, there are two types of ARL, namely in-control ARL (ARL_0_) and out-of-control ARL (ARL_1_). Here, ARL_0_ is the ARL value obtained when the actual process is under control. If the probability of a Type I error is set as α, ARL_0_ can also be defined as follows:

$$ARL_{0}=\frac{1}{\alpha }$$

Next, ARL_1_ was obtained when the process was in an out-of-control state. If the probability of a Type II error is defined as β, ARL_1_ can also be defined as follows:

$$ARL_{1}=\frac{1}{1-\beta }$$

In performance evaluation, a simulation study was conducted to illustrate the simultaneous control chart or Max Chart with the SRS scheme and the Improved Bootstrap Max chart control chart under the RSS scheme. Simulation studies were conducted under the assumption that the data for the SRS scheme comes from a normal distribution with $\mu_{0}=0$ and $\sigma_{0}=1$. For the RSS scheme, it is assumed to come from random variable *X* and its concomitant variable *Y*, which are distributed as $\left(\begin{array}{c} X\\ Y \end{array}\right)∼N\left[\left(\begin{array}{c} 0\\ 0 \end{array}\right),\left(\begin{array}{cc} 1 & \rho \\ \rho & 1 \end{array}\right)\right]$ for $h^{2}$ with combinations of ρ=0,3;0,5;0,9. Each sampling scheme (SRS and RSS) was generated with a subgroup size of 5. For the improved algorithm, the values δ=1 and ε=5 are set. For the bootstrap algorithm, n_b_=1000 was determined. Next, to obtain the value of ARL_1_, we perform a shift in the mean ($\mu_{x}$= $\mu_{x}+d\;\sigma_{x}$), a shift in the variance ($\sigma_{x}$= $\sigma_{x}\left(1+d\right)$), and a simultaneous shift in mean and variance ($\mu_{x}$= $\mu_{x}+d\;\sigma_{x}$ and $\sigma_{x}$= $\sigma_{x}\left(1+d\right)$). The value of *d* was set to 0.1.

### Evaluation findings

In this section, the results of the simulation study conducted to evaluate the performance of the proposed control chart are presented. Table 3 presents the results of the ARL_0_ and ARL_1_ values for shifts in mean only for the simultaneous Max chart based on SRS and the Improved Bootstrap Max chart based on RSS. Table 4 presents the results of ARL_0_ and ARL_1_ values for shifts in variance only for the simultaneous Max chart based on SRS and the Improved Bootstrap Max chart based on RSS. Table 5 provides information on the ARL_0_ and ARL_1_ values for shifts in mean and variance for the conventional Max chart based on SRS and the Improved Bootstrap Max chart based on RSS.

**Table 3:** Values of ARL_0_ and ARL_1_ for mean shifts with *n* = 5.

shift mean	SRS	RSS		
		ρ=0.3	ρ=0.5	ρ=0.9
0	367.076	370.633	381.433	376.997
0.1	318.124	330.68	321.243	290.268
0.2	236.66	228.586	225.375	155.112
0.3	151.311	139.724	128.763	69.334
0.4	89.381	85.683	70.567	34.862
0.5	52.825	48.176	42.688	18.017
0.6	32.557	27.949	22.934	9.632
0.7	20.202	18.131	14.871	5.491
0.8	12.986	11.015	9.95	3.711
0.9	8.654	7.528	6.515	2.529
1	6.151	5.19	4.419	1.972
1.1	4.226	3.988	3.414	1.563
1.2	3.28	3.123	2.542	1.352
1.3	2.697	2.409	2.062	1.185
1.4	2.21	1.992	1.759	1.1
1.5	1.828	1.675	1.48	1.063
1.6	1.544	1.45	1.318	1.027
1.7	1.399	1.314	1.226	1.011
1.8	1.255	1.224	1.138	1.005
1.9	1.179	1.145	1.073	1.001
2	1.102	1.109	1.056	1
2.1	1.075	1.046	1.026	1.001
2.2	1.054	1.034	1.02	1
2.3	1.027	1.017	1.011	1
2.4	1.017	1.012	1.004	1
2.5	1.008	1.006	1.001	1
2.6	1.001	1.002	1.001	1
2.7	1.003	1.001	1	1
2.8	1	1	1	1
2.9	1	1	1	1
3	1	1	1	1

**Table 4:** Values of ARL_0_ and ARL_1_ for variance shift with *n* = 5.

shift variance	SRS	RSS		
		ρ=0.3	ρ=0.5	ρ=0.9
1	372.008	370.587	368.99	378.132
1.1	150.048	140.661	143.083	146.052
1.2	63.969	58.269	56.416	56.853
1.3	28.779	28.425	27.276	24.449
1.4	16.257	15.584	14.888	12.71
1.5	9.633	9.546	9.297	8.771
1.6	6.958	6.582	6.564	5.794
1.7	5.062	4.949	4.639	4.192
1.8	3.863	3.842	3.551	3.28
1.9	3.251	2.921	2.977	2.497
2	2.566	2.626	2.558	2.253
2.1	2.313	2.253	2.166	1.969
2.2	2.064	1.996	1.998	1.731
2.3	1.841	1.788	1.688	1.691
2.4	1.698	1.727	1.631	1.503
2.5	1.669	1.58	1.549	1.411
2.6	1.506	1.459	1.466	1.337
2.7	1.438	1.421	1.382	1.279
2.8	1.39	1.365	1.375	1.232
2.9	1.308	1.287	1.294	1.208
3	1.259	1.262	1.273	1.182
3.1	1.243	1.223	1.203	1.133
3.2	1.206	1.198	1.211	1.135
3.3	1.163	1.182	1.162	1.103
3.4	1.15	1.163	1.162	1.093
3.5	1.132	1.144	1.113	1.088
3.6	1.127	1.113	1.112	1.066
3.7	1.093	1.097	1.105	1.059
3.8	1.122	1.098	1.101	1.065
3.9	1.11	1.088	1.087	1.039
4	1.064	1.071	1.075	1.039

**Table 5:** Values of ARL_0_ and ARL_1_ for mean and variance shift with *n* = 5.

shift mean	shift variance	d	SRS	RSS		
				ρ=0.3	ρ=0.5	ρ=0.9
0	1	0	378.723	379.676	379.885	385.556
0.1	1.1	0.1	141.767	133.923	135.584	118.782
0.2	1.2	0.2	50.837	46.181	42.624	36.491
0.3	1.3	0.3	22.463	20.428	19.899	14.416
0.4	1.4	0.4	10.903	10.4	9.929	7.643
0.5	1.5	0.5	6.581	6.496	6.148	4.451
0.6	1.6	0.6	4.518	4.435	4.132	3.024
0.7	1.7	0.7	3.37	3.319	3.016	2.286
0.8	1.8	0.8	2.639	2.647	2.472	1.926
0.9	1.9	0.9	2.131	2.031	2.112	1.578
1	2	1	1.906	1.92	1.779	1.397
1.1	2.1	1.1	1.747	1.669	1.626	1.334
1.2	2.2	1.2	1.573	1.559	1.49	1.27
1.3	2.3	1.3	1.445	1.428	1.366	1.176
1.4	2.4	1.4	1.377	1.311	1.24	1.112
1.5	2.5	1.5	1.309	1.221	1.22	1.086
1.6	2.6	1.6	1.239	1.205	1.195	1.082
1.7	2.7	1.7	1.208	1.199	1.149	1.054
1.8	2.8	1.8	1.151	1.176	1.109	1.041
1.9	2.9	1.9	1.144	1.116	1.102	1.035
2	3	2	1.105	1.099	1.08	1.029
2.1	3.1	2.1	1.113	1.071	1.088	1.019
2.2	3.2	2.2	1.071	1.064	1.075	1.019
2.3	3.3	2.3	1.063	1.05	1.06	1.017
2.4	3.4	2.4	1.05	1.05	1.046	1.013
2.5	3.5	2.5	1.052	1.039	1.031	1.013
2.6	3.6	2.6	1.027	1.039	1.03	1.006
2.7	3.7	2.7	1.053	1.032	1.02	1.007
2.8	3.8	2.8	1.03	1.028	1.022	1.007
2.9	3.9	2.9	1.027	1.025	1.019	1.002
3	4	3	1.014	1.022	1.015	1.005

**Fig. 1:**
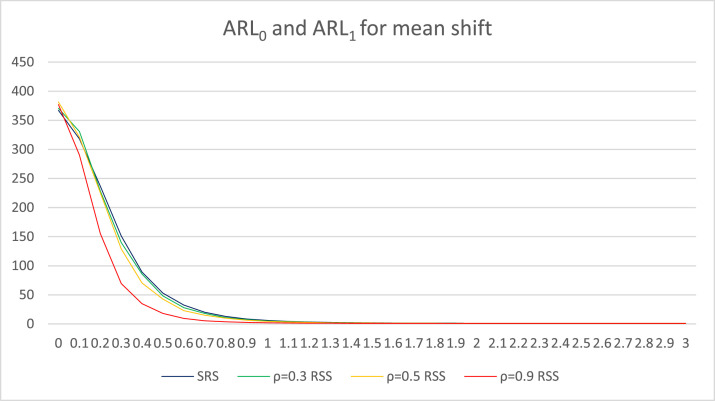
ARL graphics for mean shifts with *n* = 5.

**Fig. 2:**
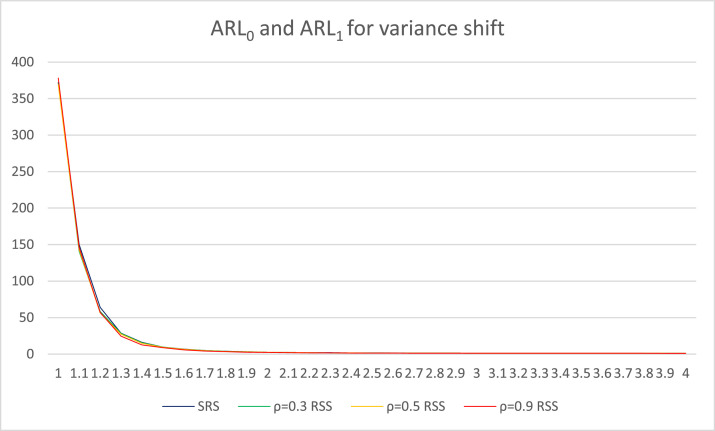
ARL graphics for variance shifts with *n* = 5.

**Fig. 3:**
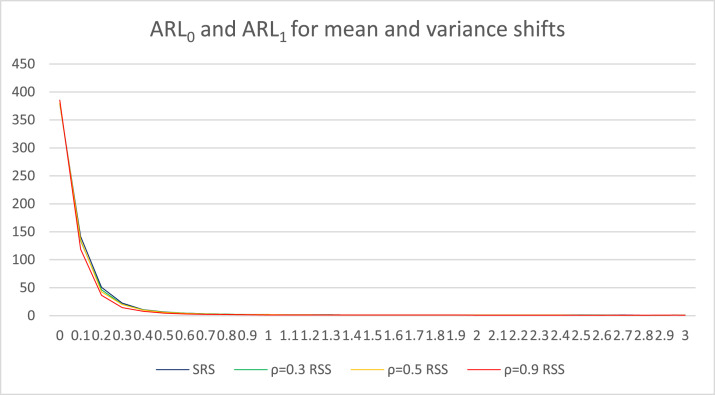
ARL graphics for mean and variance shifts with *n* = 5.

In order to assist analyzing the changes in the ARL values due to shifts, these changes are illustrated in a line chart. Fig. 1 graphically depicts the changes in ARL values for mean shifts. Fig. 2 presents information about changes in ARL values for variance shifts. And Fig. 3 illustrates the decreasing ARL values as a mean and variance shift.

In Table 3, the ARL values for mean shifts are presented. The first column presents information on the initial value of the mean followed by a mean shift that changes by *d* = 0.1. The second column presents information on the ARL value from the conventional Max Chart based on SRS. In the third, fourth, and fifth columns, information on the ARL values from the Max Chart based on RSS is presented, with the correlation between the concomitant variables and the monitored objects being ρ=0.3, ρ=0.5, and ρ=0.9, respectively. To help understand the information presented in Table 3, the ARL values in Table 3 are also visualized in Fig. 1. In Fig. 1, the blue line illustrates the change in ARL values as the mean value shifts from the conventional Max chart based on SRS. The change in ARL values as the mean value shifts in the RSS-based Max control chart is depicted with a green line for ρ=0.3, a yellow line for ρ=0.5, and a red line for ρ=0.9.

From the information in Table 3, when the mean has not shifted or the mean shift = 0, the ARL_0_ value of each simulated control chart is around 370. Next, a better-performing control chart has a smaller ARL_1_ value as the mean shift. The performance results of the evaluated control charts can also be observed in Fig. 1. From Fig. 1, it can be seen that the ARL_1_ value of the Improved Bootstrap Max chart based on RSS with ρ =0.9 drops the fastest as the mean shifts. This is followed by the Improved Bootstrap Max chart based on RSS with ρ =0.5 and ρ =0.3. The slowest decrease is shown by the conventional Max chart based on SRS. The information in Table 3 and Fig. 1 shows that the performance of the Improved Bootstrap Max chart based on RSS with ρ=0.3, 0.5, and 0.9 is better than the conventional Max chart based on SRS because the ARL_1_ value of the Improved Bootstrap Max chart based on RSS with ρ=0.3, 0.5, and 0.9 is always smaller than the conventional Max chart based on SRS when a mean shift occurs. This means that the Improved Bootstrap Max chart based on RSS with ρ=0.3, 0.5, and 0.9 is better at detecting the mean shift than the conventional Max chart based on SRS. The findings of this study support the research by Muttlak and Al-Sabah in 2003, which stated that the control chart based on RSS outperforms the control chart based on SRS in detecting Mean shifts [9].

In Table 4, the ARL values for the variance shift are displayed. The initial column provides details about the starting value of the variance, followed by a shift in the variance that changes by *d* = 0.1. The second column outlines the ARL values derived from the conventional Max Chart based on SRS. The third, fourth, and fifth columns present the ARL values from the Max Chart based on RSS, with the correlation between the concomitant variables and the monitored objects set at ρ=0.3, ρ=0.5, and ρ=0.9, respectively. To facilitate understanding of the information shown in Table 4, the ARL values are also visualized in Fig. 2, which offers a clear and concise representation of the data. In Fig. 2, the blue line indicates the changes in ARL values as the mean shifts in the conventional Max Chart based on SRS. Furthermore, the changes in ARL values resulting from the mean shifts in the Improved Bootstrap Max Chart based on RSS are illustrated with a green line for ρ=0.3, a yellow line for ρ=0.5, and a red line for ρ=0.9.

According to Table 4, when there is no variance shift (i.e., shift variance = 0), the ARL_0_ value for each simulated control chart is approximately 370. A more effective control chart demonstrates a lower ARL_1_ value as the variance shifts. As demonstrated in Fig. 2, the ARL_1_ value of the Improved Bootstrap Max chart based on RSS with ρ =0.9 exhibits the most rapid decline as the variance shifts. The subsequent chart is the Improved Bootstrap Max chart based on RSS with ρ =0.5 and ρ =0.3. The conventional Max chart based on SRS demonstrates the slowest decrease. As indicated in Table 4 and Fig. 2, when a variance shift occurs, the performance of the Improved Bootstrap Max Chart based on the RSS with ρ=0.3, 0.5, and 0.9 surpasses that of the conventional Max chart based on SRS. This is evidenced by the consistently smaller ARL_1_ values for the Improved Bootstrap Max Chart based on the RSS with ρ=0.3, 0.5, and 0.9 compared to the conventional Max chart based on SRS, indicating that the former is more adept at detecting variance shifts. The results of this study are consistent with the findings of Abbas et al. in 2003 [11], which found that the RSS-based control chart outperforms the SRS-based control chart for monitoring variability.

Table 5 presents the ARL values for simultaneous shifts in mean and variance. The first column presents the initial values of the mean and variance, followed by the simultaneous shifts in the mean and variance that change by *d* = 0.1. The second column presents information on the ARL values from the conventional Max chart based on SRS. In the third, fourth, and fifth columns, information on the ARL values from the Improved Bootstrap Max chart based on RSS is presented, with the correlation between the concomitant variables and the monitored objects being ρ=0.3, ρ=0.5, and ρ=0.9, respectively. The ARL values in Table 5 are also visualized in Fig. 3 to understand the information that is presented in Table 5. In Fig. 3, the blue line illustrates the change in ARL values as the mean and variance value shifts from the conventional Max chart based on SRS. The change in ARL values as the mean and variance value shifts from the Improved Bootstrap Max chart based on RSS is depicted with a green line for ρ=0.3, a yellow line for ρ=0.5, and a red line for ρ=0.9.

According to the information presented in Table 5, when the mean and variance remain unchanged (i.e., *d* = 0), the ARL_0_ value for each simulated control chart is approximately 370. Furthermore, a control chart that performs better exhibits a smaller ARL_1_ value during simultaneous shifts in both the mean and variance. As can be seen in Fig. 3, the ARL_1_ value of the Improved Bootstrap Max chart based on RSS with ρ = 0.9 decreases the fastest when there are simultaneous shifts in both the mean and variance. This is followed by the Improved Bootstrap Max chart based on RSS with ρ = 0.5 and ρ = 0.3. The conventional Max chart based on SRS shows the slowest decrease. As indicated in Table 5 and Fig. 3, the performance of the Improved Bootstrap Max chart based on RSS, with ρ values of 0.3, 0.5, and 0.9, outperforms the conventional Max chart based on SRS when there are simultaneous shifts in both parameters. Specifically, the ARL_1_ values for the Improved Bootstrap Max chart based on RSS are consistently lower than the conventional Max chart based on SRS. This demonstrates that the Improved Bootstrap Max chart using RSS with ρ=0.3, 0.5, and 0.9 is more effective in detecting simultaneous shifts in both mean and variance compared to its SRS counterpart. This finding shows that the control chart based on RSS not only outperforms the control chart based on SRS for partial monitoring of mean and variance [9,11] but also outperforms the control chart based on SRS for simultaneous monitoring of mean and variance.

Based on the previous explanation, several points can be discussed. First, from the performance evaluation results, it can be concluded that the proposed control chart provides a consistent ARL_0_ value of approximately 370 for α=0.0027. This indicates that the proposed control chart can precisely estimate the UCL value. This is due to the improved bootstrap algorithm is specifically designed to obtain the UCL according to the specified α value. The second point is that the proposed control chart can effectively detect shifts in the mean or variance of the monitored objects, either partially or simultaneously. In particular, under strong ρ conditions. This is because the information obtained from the sample in the RSS scheme can more accurately represent the population conditions than in the SRS scheme. Therefore, we can conclude that the simultaneous univariate control chart with the improved bootstrap algorithm based on the RSS scheme performs better than the conventional Max chart based on SRS in detecting shifts.

### Practical scenario

**Fig. 4:**
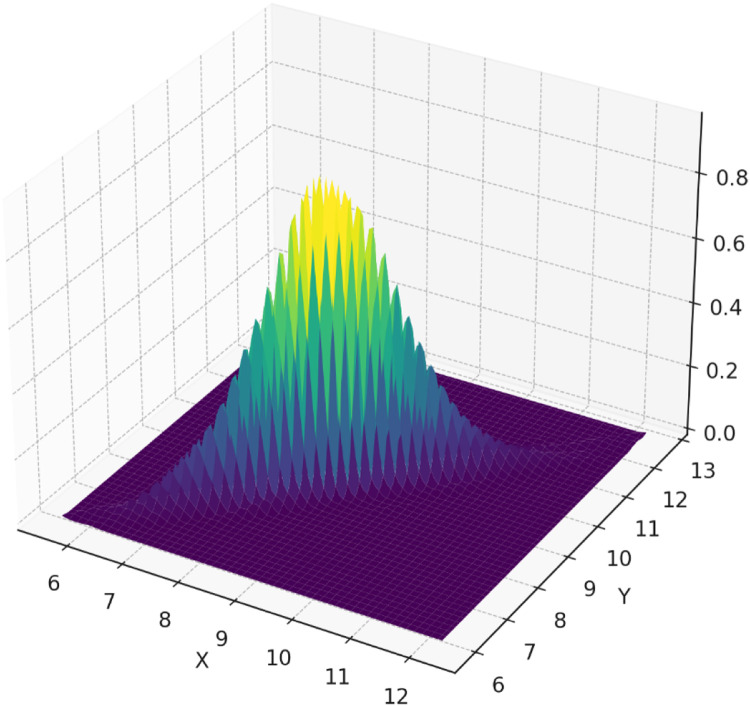
The probability density plot of variables X (RVP Lab) and Y (RPV Field).

In this section, an application example is presented to illustrate the use of the proposed Improved Bootstrap Max Chart based on the RSS scheme. An example of an application is the monitoring of the Reid Vapor Pressure (RVP) of gasoline [26]. Gasoline samples were collected from gas stations for RVP monitoring. The RVP of gasoline can be measured in the laboratory or at a gas station. Laboratory measurements yield more accurate RVP values but are more expensive than gas station measurements; therefore, laboratory RVP values are recommended. In this case, RSS can be used as a sampling method to monitor RVP values. The cheaper measurement at the gas station (RVP Field or Y) can be used as a concomitant variable for gasoline sampling in the laboratory. Concomitant variables were used to assist the sampling process using the RSS. The RVP measurement results from this laboratory can be referred to as the RVP Lab or X. From the parameters taken from Chen et al. [26], the RVP Field data for the SRS scheme comes from a normal distribution with $\mu_{0}=8.649$ kPa and $\sigma_{0}=0.542$ kPa. The RVP Lab and RVP Field data for RSS taken from a total of 1750 or 70 % in-control data which are distributed as $\left(\begin{array}{c} X\\ Y \end{array}\right)∼N\left[\left(\begin{array}{c} 8.649\\ 8.642\; \end{array}\right),\left(\begin{array}{cc} 0.542 & 0.699\\ 0.699 & 0.497 \end{array}\right)\right]$ and 750 or 30 % out-of-control data which are distributed as $\left(\begin{array}{c} X\\ Y \end{array}\right)∼N\left[\left(\begin{array}{c} 8.649\\ 8.642 \end{array}\right),\left(\begin{array}{cc} 1.528 & 1.445\\ 1.445 & 1.452 \end{array}\right)\right]$. The probability density plot of in-control data X (RVP Lab) and Y (RPV Field) is given in Fig. 4. From the data, sampling was conducted using RSS and SRS methods, where each sampling method produced 100 sample subgroups with a size of 5 for each subgroup. The data were collected from one observation subgroup on X (RVP Lab) using the RSS scheme as follows:

Step 1: Select 25 units of data from the gasoline sample that has been measured for the Y value (RVP Field) using the SRS scheme and group them into 5 sets with each set containing 5 units.
Step 2: Sort the gasoline samples based on the value of Y (RPV Field) in each set.
Step 3: To obtain the sample that will be taken to the laboratory or sample X (RPV Lab), we select a sample from the first set that has the lowest Y value (RPV Field). In the same manner, we take a sample from the second set that has the second lowest Y value (RPV Field). This process was continued until the fifth sample, which is the sample with the highest Y value (RPV Field) in the fifth set.

In order to demonstrate the accuracy of the Improved Bootstrap Max Chart based on the RSS compared to the conventional Max Chart based on SRS, the performance of each control chart is evaluated with a confusion matrix as shown in Table 6. Accuracy in monitoring organic water can be divided into two types, namely:

1. True Positives (TP) is the amount of data in control that is detected in control.
2. True Negative (TN) is the amount of data out of control that is detected as out of control.

**Table 6:** Confusion matrix for monitoring water quality.

	detected	
	In control	Out of control
in Control Data	True Positives (TP)	False Negative (FN)
out of control data	False Positive (FP)	True Negative (TN)

Furthermore, to see the accuracy level, the hit rate is used which can be calculated as follows:

$$\text{Hit}\;\text{Rate}=\;\frac{\text{TP}+\text{TN}}{\text{TP}+\text{TN}+\text{FP}+\text{FN}}$$

**Fig. 5:**
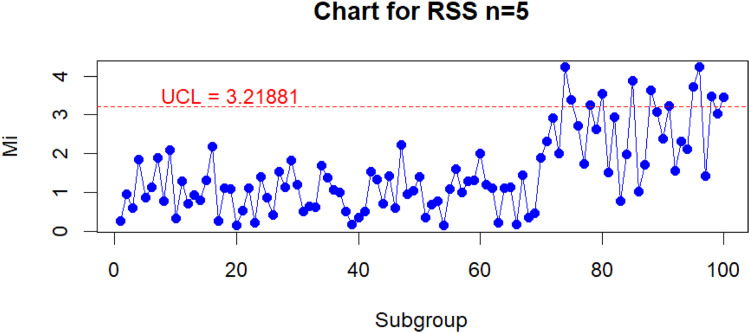
Monitoring RVP Lab values using the improved bootstrap max chart under the RSS scheme.

**Fig. 6:**
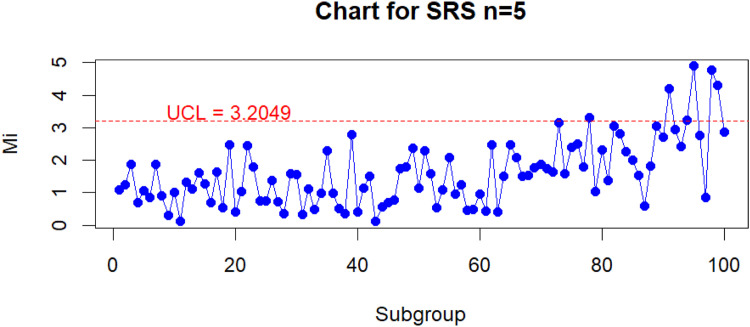
Monitoring RVP Lab values using the conventional simultaneous max chart control chart.

The results of monitoring RPV Lab using the Improved Bootstrap Max Chart based on the RSS is shown in Fig. 5 and The results of monitoring RPV Lab using the conventional Max chart control chart based on the SRS is shown in Fig. 6, with the confusion matrix table listed in Table 7, Table 8. The Hit rate results are also avalaible in Table 9 for both of control charts.

**Table 7:** The confusion matrix of Max Chart under RSS Scheme.

	detected	
	In control	Out of control
in control data	70	0
out of control data	19	11

**Table 8:** The confusion matrix of Max Chart under SRS Scheme.

	detected	
	In control	Out of control
in control data	70	0
out of control data	24	6

**Table 9:** The hit rate of control charts.

	Hit Rate
SRS	0.76
RSS	0.81

Based on the information from Fig. 5, Fig. 6, it can be seen that the Improved Bootstrap Max Chart based on the RSS can detect shifts more quickly, starting at point 74, whereas the conventional Max chart control chart based on the SRS can only detect shifts at point 78. Additionally, with reference to the confusion matrix table that can be found in Table 7, Table 8., the Improved Bootstrap Max Chart based on the RSS can detect 11 shift points, outperforming the conventional Max chart control chart based on the SRS, which can only detect 6 shift points in the RVP Lab value monitoring process. Based on the hit rate comparison in Table 9, The Improved Bootstrap Max chart based on RSS with a hit rate of 0.83 outperforms the conventional Max chart control chart based on RSS with a hit rate of 0.76. This confirms that when monitoring the RVP value process, the Improved Bootstrap Max Chart based on the RSS exhibits superior performance compared to the conventional Max chart control chart based on the SRS. Overall, the results of this data application strongly support the selection of the Improved Bootstrap Max Chart based on the RSS as an alternative to a more sensitive and effective control chart in detecting out-of-control conditions compared to the conventional Max chart control chart based on the SRS.

## Limitations

The data used to evaluate the proposed control chart were generated from Normal distribution. It is due to the simultaneous univariate Shewhart control chart, also known as the Max Chart, is assumed to monitor data that follows a normal distribution. The assumption of normality is linked to the process of transforming the statistic of the mean and variance. This transformation process is important for simultaneously monitoring the mean and variance in a single control chart.

## CRediT authorship contribution statement

**Sukma Adi Perdana:** Conceptualization, Methodology, Software, Writing – original draft, Visualization. **Muhammad Mashuri:** Conceptualization, Methodology, Writing – review & editing, Validation, Supervision. **Muhammad Ahsan:** Conceptualization, Methodology, Writing – review & editing, Validation, Supervision.

## Declaration of competing interest

The authors declare that they have no known competing financial interests or personal relationships that could have appeared to influence the work reported in this paper.

## Data Availability

Data will be made available on request.
